# Supplementary material for: ICAM-1/CD18-mediated sequestration of parasitized phagocytes in cortical capillaries promotes neuronal colonization by Toxoplasma gondii
Source: Nat Commun. 2025 Apr 14;16:3529. doi: 10.1038/s41467-025-58655-z (PMC11997185; doi:10.1038/s41467-025-58655-z)
Supplement: Supplementary file 1 — Supplementary Information [file 41467_2025_58655_MOESM1_ESM.pdf]

Supplementary information

ICAM-1/CD18-mediated sequestration of parasitized phagocytes in cortical capillaries promotes neuronal colonization by *Toxoplasma gondii*

Matias E. Rodriguez[1], Ali Hassan[1], Nikolaos Linaroudis[1], Felix Harryson-Oliveberg[1], Arne L. ten Hoeve[1], Antonio Barragan[1, *]

[1]Department of Molecular Biosciences, The Wenner-Gren Institute, Stockholm University, 10691 Stockholm, Sweden

Supplementary data tables

Name	Target gene	Sequence
TgB1_F	*Tg b1*	GCATTGCCCGTCCAAACT
TgB1_R	*Tg b1*	AGACTGTACGGAATGGAGACGAA
Vcam-1_F	*Vcam1*	GTG ACT CCA TGG CCC TCA CTT
Vcam-1_R	*Vcam1*	CGT CCT CAC CTT CGC GTT TA
Icam-1_F	*Icam1*	CAA TTT CTC ATG CCG CAC AG
Icam-1_R	*Icam1*	CTG GAA GAT CGA AAG TCC GG
Sele_F	*Elam1*	CCC TGC CCA CGG TAT CAG
Sele_R	*Elam1*	ACG TGC ATG TCG TGT TCCA
Timp1_F	*Timp1*	GCAACTCGGACCTGGTCATAA
Timp1_R	*Timp1*	CGCTGGTATAAGGTGGTCTCG
CCL-2_F	*Ccl2*	CATCCACGTGTTGGCTCA
CCL-2_R	*Ccl2*	GATCATCTTGCTGGTGAATGAGT
CCL-5_F	*Ccl5*	ATATGGCTCGGACACCACTC
CCL-5_R	*Ccl5*	TTCGAGTGACAAACACGACTG
GAPDH_F	*Gapdh*	TGACCTCAA CTACATGGTCTACA
GAPDH_R	*Gapdh*	CTTCCCATT CTCGGCCTTG
HPRT_F	*Hrpt*	CCC AGC GTC GTG ATT AGC
HPRT_R	*Hrpt*	GGA ATA AAC ACT TTT TCC AAA TCC

Table S1. Primer sequences for qPCR

Supplementary figures

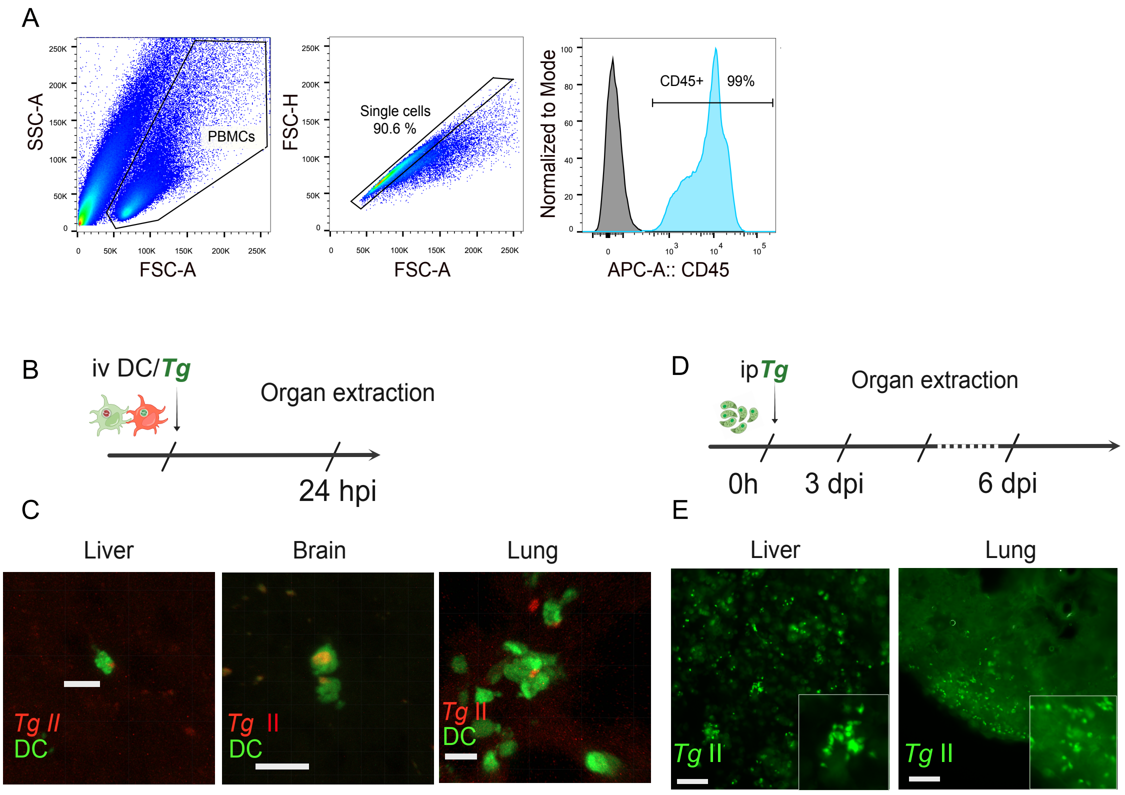

Figure S1. Flow cytometry gatings, sequestration and parasite load in lungs and liver

A. Representative flow cytometry plots show CD45 expression on PBMCs in control conditions. PBMCs were identified based on scatter-A/side scatter-A (left panel), followed by single cell (middle panel) analysis of CD45 expression (right panel, MFI normalized to mode). Mice were injected iv with 0.4 mg/kg anti-CD45-647. After 10 min, blood was collected.

B. CFSE pre-labelled DCs were challenged *in vitro* with *T. gondii* (type II ME49-RFP) to obtain a DC infection frequency of ~50%. Infected DCs ($40x10^6$ DCs / $20x10^6$ cfu *Tg*) were inoculated iv in mice and organs were collected 24 hpi.

C. Representative micrographs show *T. gondii* (ME49-RFP)-infected DCs ($CFSE^+$, RFP^+) in liver, brain and lung. Scale bar: 10 µm.

D. Experimental set up. Freshly egressed GFP-expressing *T. gondii* (*Tg*) tachyzoites (PRU-GFP, $2x10^5$ cfu) were inoculated ip in mice and organs were collected 3 or 6 dpi.

E. Representative micrographs show *T. gondii* (PRU-GFP) foci in liver, brain and lung. Scale bar: 50 µm.

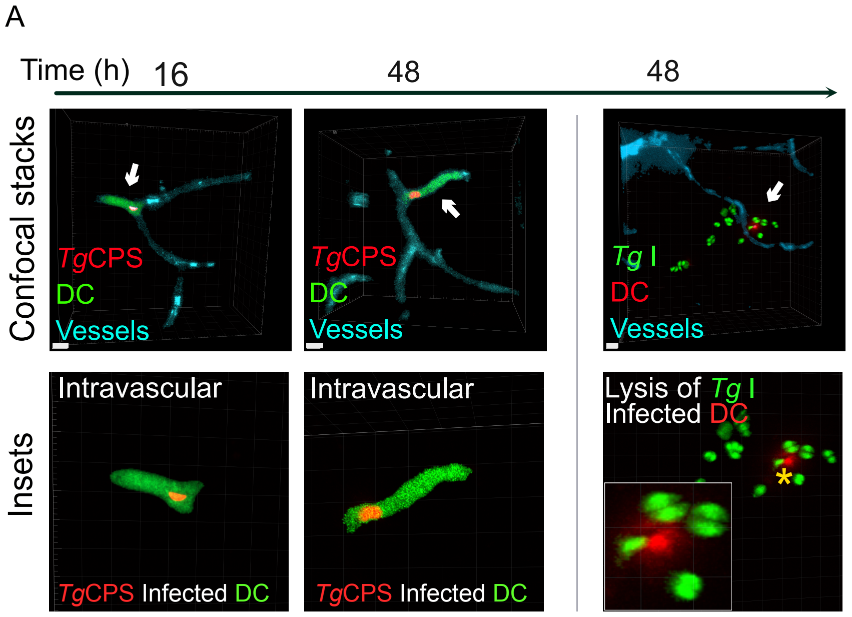

Figure S2. Sequestration in the absence of replication

A. CMTMR or CFSE pre-labelled DCs were challenged *in vitro* with *T. gondii* RH-WT (GFP^{+}, MOI 1) or RH-CPS (mCherry^{+}, MOI 1), respectively, to obtain a DC infection frequency of ~50%. Infected DCs ($20x10^{6}$ DCs / $10x10^{6}$ cfu *Tg*) were inoculated in the ICA and brains collected 16 or 48 hpi. Confocal micrographs show intravascularly located (Evans blue, cyan) RH-CPS infected DCs (CFSE^{+}; mCherry^{+}) at 16 and 48 hpi (left panels) and extravascular RH-WT tachyzoites (GFP^{+}) at 48 hpi (right panel). Arrows indicate infected DCs. Asterisk indicates DC CMTMR^{+} remnant ("*ghost", red*). Scale bars: 10 µm.

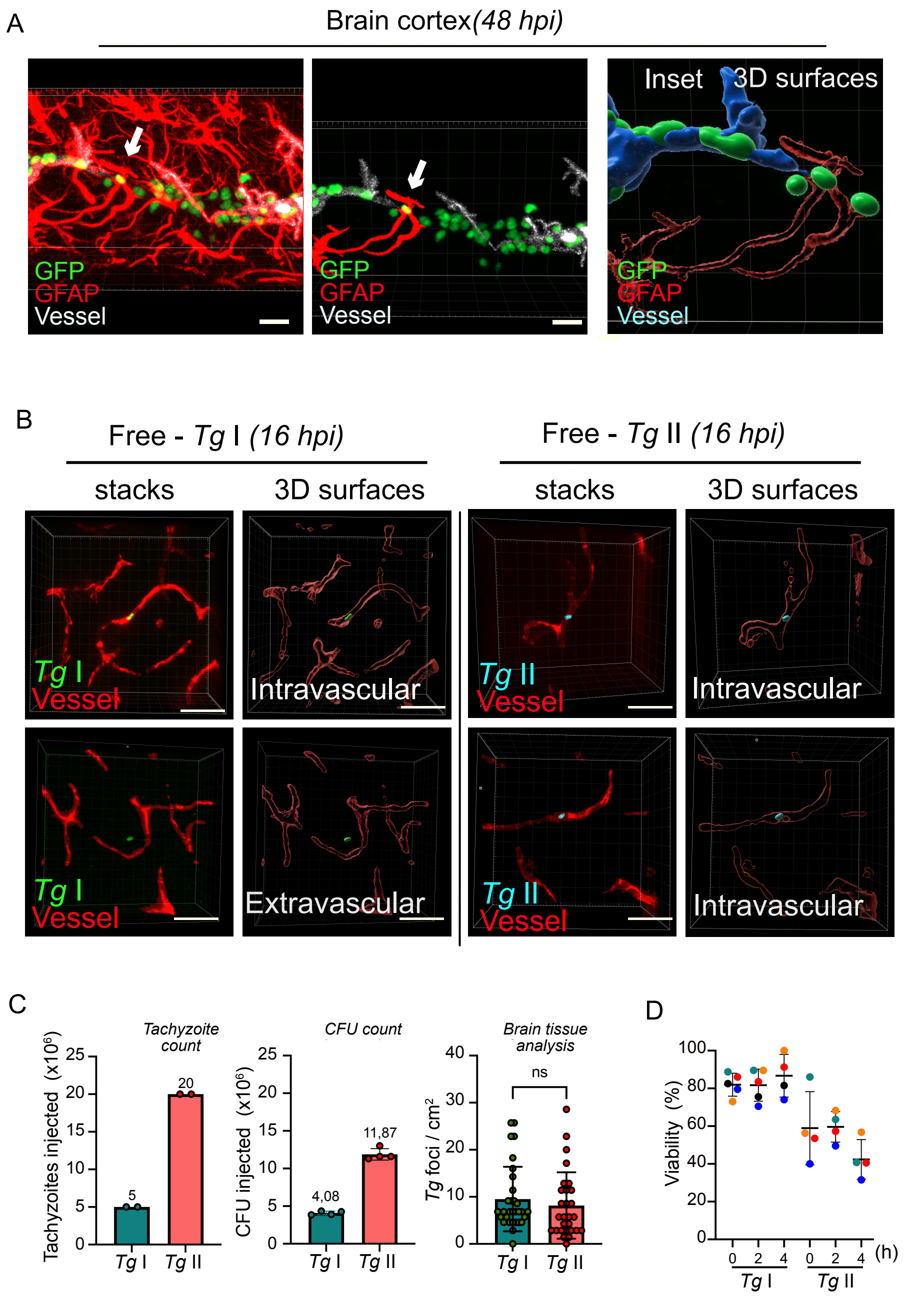

Figure S3. Association of *T. gondii* with astrocytes and vascular/parenchymal localization after inoculation of free (extracellular) tachyzoites and viability tests

A. Infected CMTMR pre-labelled DCs ($20x10^6$ DCs / $10x10^6$ cfu *Tg*) were inoculated in the ICA and brains extracted 48 hpi. Confocal micrographs (left panels) show the localization of RH tachyzoites (GFP^+, green) and astrocytes ($GAFP^+$, red) in relation to the vascular marker Evans blue (grey). Arrows indicate infected $GAFP^+$ cell magnified in inset (right panel). Right panel: Confocal 3D surface analysis shows $GAFP^+$ cell-associated tachyzoite. Scale bar: 10 µm.

B. Confocal micrographs and corresponding 3D surfaces illustrate intravascular and extravascular localization of tachyzoites (type I RH, GFP^+ green and type II ME49, RFP^+, cyan) in relation to the vascular marker Evans blue (red). $20x10^6$ cfu of freshly egressed tachyzoites were inoculated in the ICA and brains were collected 16 hpi. Scale bar: 50 µm.

C. Bar graphs show numbers of type I and II injected in order to reach comparable cerebral parasite loads. $5x10^6$ type I (RH) tachyzoites and $20x10^6$ type II (PRU) tachyzoites were injected in the ICA. Plaquing assays yielded 4 $x10^6$ type I (RH) colony-forming units (cfu) and $12x10^6$ type II (PRU) cfu. Tissue analyses yielded comparable numbers of foci in brain tissue. For each condition, data are from 2 independent experiments performed in duplicate and representative of multiple experiments with variable doses injected (n= 2 mice). 2-tailed Mann-Whitney U-test. ns: non-significant.
D. Viability of type I (RH) and type II (PRU, ME49) tachyzoites at indicated timepoints determined by plaquing assays. Data are expressed as mean ± SEM from 4-5 independent experiments. For each experiment, datapoints are color-coded accordingly.

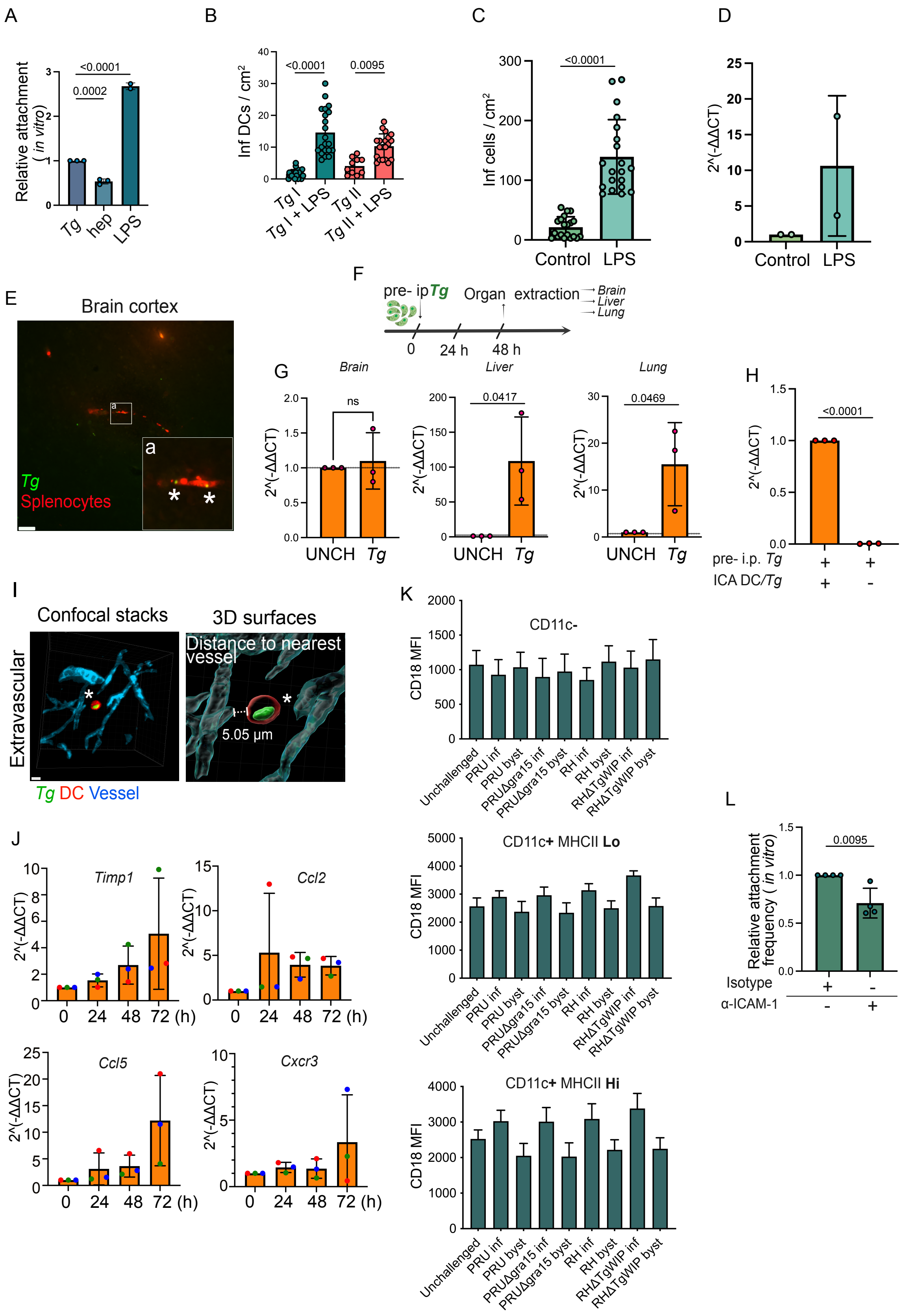
A
Relative attachment (in vitro)
<0.0001
0.0002
Tg
hep
LPS
B
Inf DCs / cm²
<0.0001
0.0095
Tg I
Tg I + LPS
Tg II
Tg II + LPS
C
Inf cells / cm²
<0.0001
Control
LPS
D
2^(-ΔΔCT)
Control
LPS
E
Brain cortex
a
Tg
Splenocytes
F
pre- ip Tg
Organ extraction
Brain
Liver
Lung
0
24 h
48 h
G
Brain
Liver
Lung
2^(-ΔΔCT)
ns
0.0417
0.0469
UNCH
Tg
H
2^(-ΔΔCT)
<0.0001
pre- i.p. Tg
ICA DC/Tg
I
Confocal stacks
3D surfaces
Distance to nearest vessel
5.05 µm
Extravascular
Tg DC Vessel
J
Timp1
Ccl2
Ccl5
Cxcr3
2^(-ΔΔCT)
0
24
48
72 (h)
K
CD11c-
CD11c+ MHCII Lo
CD11c+ MHCII Hi
CD18 MFI
Unchallenged
PRU inf
PRU byst
PRUΔgra15 inf
PRUΔgra15 byst
RH inf
RH byst
RHΔTgWIP inf
RHΔTgWIP byst
L
Relative attachment frequency (in vitro)
0.0095
Isotype
α-ICAM-1

Figure S4. Role of the inflammatory response on sequestration

A. Graph shows the relative attachment frequency of *T. gondii* type II PRU-infected DCs to polarized brain endothelial cells (bEnd.3) *in vitro,* in control (*Tg*), heparin or LPS pre-treatment conditions. Data expressed as mean (± SEM) from three independent experiments.

B. Graph shows the absolute number (mean ± SEM) of *T. gondii* type I RH-GFP and type II ME49-RFP-infected DCs per cm^2 of cortical tissue at 16 hpi. Mice were inoculated in the ICA with $20x10^6$DCs /$10x10^6$ cfu *Tg* in control (*Tg*) and LPS pre-treated conditions. Data are from 32 (DC/*Tg*I), 30 (DC/*Tg*I + LPS), 30 (DC/*Tg*II) and 29 (DC/*Tg*II + LPS) cortical sections per condition from three independent experiments (n=3 mice per condition).

C, D. Graph shows the absolute number (mean ± SEM) of *T. gondii* (type II, PRU-GFP)-infected splenocytes per cm^2 of cortex tissue at 16 hpi (C) and the relative expression (qPCR) of *TgB1* gene in brain tissue (D), respectively. Mice were inoculated in the ICA with $20x10^6$ DCs/$10x10^6$ cfu *Tg* in control (*Tg*) and LPS pre-treatment conditions. Data from 20 cortical sections per condition from 2 independent experiments (n= 2 mice).

E. Representative micrographs show *T. gondii* (type II, PRU-GFP)-infected splenocytes ($CMTMR^+$, GFP^+) in cortical sections of LPS pre-treated mice at 16 hpi. Insets (a) shows magnification of the white box and asterisks indicate infected splenocytes ($CMTMR^+$ GFP^+, red/green). Scale bar: 20 µm.

F. Experimental set up. Mice were inoculated ip with $2x10^5$ cfu of freshly egressed tachyzoites (type II, ME49-RFP) or control medium. After 48 h, brain, liver and lung were collected.

G. Graphs show the relative expression (qPCR) of *TgB1* gene (mean± SEM) in brain, liver and lung tissues of control (unchallenged) or infected mice (*Tg*) at 48 hpi. Data from three independent experiments (n= 3 mice).

H. Graph shows the relative expression of *TgB1 gene* (mean± SEM) in brain tissue of pre- ip infected mice (pre-ip *Tg*) and pre- ip infected mice plus inoculation of infected DCs in the ICA (pre- ip *Tg* + ICA DC/*Tg*). Mice were inoculated ip with $2x10^5$ cfu freshly egressed tachyzoites (ME49-RFP, pre-ip *Tg*). After 48h, infected DCs ($10x10^6$ DCs/$5x10^6$ cfu Tg, PRU-GFP) were inoculated in the ICA (ICA DC/*Tg*) and brains collected 1 hpi. Data from three independent experiments (n= 3 mice).

I. Confocal micrographs and corresponding 3D surfaces show the extravascular localization (Evans blue, cyan) of *T. gondii* (PRU-GFP)-infected DC ($CMTMR^+$ GFP^+, red/green) of LPS pre-treated mice. Asterisks indicate infected $CMTMR^+$ cells. Distance to the nearest vessel is shown. Scale bars: 10 µm.

J. Relative mRNA expression (qPCR) of *Timp1* (TIMP-1), *Ccl2* (CCL2), *Ccl5* (CCL5), and *Cxcr3* (CXCR3) in brain micro-vessels. Mice were inoculated with freshly egressed RFP-expressing *T. gondii* (*Tg*) tachyzoites (ME49-RFP, $2x10^5$ cfu) or control medium and brains micro-vessels were purified at 24, 48 and 72 hpi. Data expressed as mean ± SEM from three interdependent experiments (n= 3 mice).

K. Graphs show relative expression (MFI) of CD18 in *T. gondii* WT, *ΔGRA15* or *ΔTgWIP* – infected DCs at 5 hours post challenge, assessed by flow cytometry. DCs were challenged with GFP-expressing type I RH (WT or *ΔTgWIP,* MOI 1) or type II (PRU WT or *ΔGRA15*, MOI 2) tachyzoites. Expression of CD18 (mean ± SEM) in *T. gondii*- infected (inf) and bystander (byst) $CD11c^-$, $CD11c^+$/$MHCII^{Lo}$ and $CD11c^+$/$MHCII^{Hi}$ cells is shown. Data from three interdependent experiments.

L. Bar graph shows the relative attachment frequency of *T. gondii* type II (PRU-GFP) infected DCs to polarized brain endothelial cells (bEnd.3) *in vitro*, in control (isotype) of anti-ICAM 1 pre-treatment conditions. Data are expressed as mean ± SEM from three independent experiments.

Statistical analyses: (**A, B, K)** One-way ANOVA followed by Bonferroni's multiple comparison test, (**D, G, H, L**) 2-tailed Unpaired student's t-test. (**C**) 2-tailed Mann-Whitney U-test, numeric p values are indicated, ns: non-significant.

Source data are provided as a Source Data file.

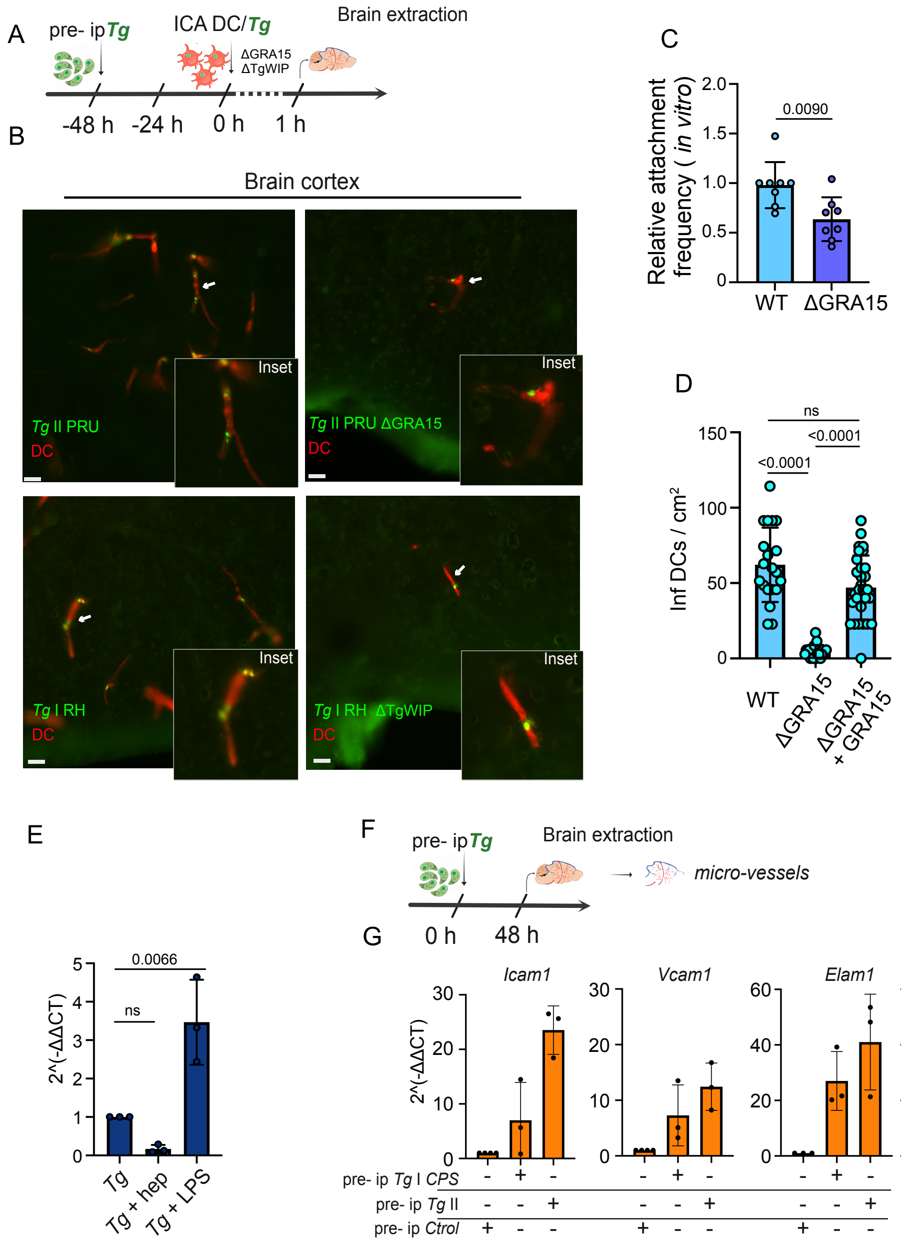

Figure S5. Localization of DCs infected with TgWIP and GRA15 mutants in the cortical microvasculature, in vitro adhesion and sequestration of DCs challenged with GRA15 mutant

A. Experimental set up. CMTMR pre-labelled DCs were challenged with GFP-expressing wild type (*WT*), *ΔGRA15* or *ΔTgWIP* tachyzoites followed by inoculation in the ICA (20x10^6 DCs / 10x10^6 cfu *Tg*). Brains were collected 16 hpi.

B. Representative micrographs show infected DCs (CMTMR$^+$, GFP$^+$) per cm^2 cortical tissue for PRU-*WT versus* PRU*ΔGRA15* (upper panels) and RH-*WT versus* RH*ΔTgWIP* (lower panels), respectively. Arrows indicate infected DCs (CMTMR$^+$ GFP$^+$, red/green) magnified in the insets. Scale bars: 20 µm.

C. Bar graph shows the relative attachment frequency of *T. gondii* type II (PRU-*WT versus* PRU*ΔGRA15*)-infected DCs to polarized brain endothelial cells (bEnd.3) *in vitro*. Data are expressed as mean (± SEM) from three independent experiments.

D. CMTMR pre-labelled DCs were challenged with GFP-expressing parental wild type (*WT,* PRU), *ΔGRA15* or *ΔGRA15* + *GRA15* tachyzoites, followed by inoculation via the ICA ($20x10^6$ DCs / $10x10^6$ cfu *Tg*). Brains were collected 16 hpi. Graphs show the absolute numbers (mean ± SEM) of infected DCs ($CMTMR^+$, GFP^+) per cm^2 cortical tissue for PRU-*WT*, PRU*ΔGRA15* and PRU*ΔGRA15* + *GRA15*. Data are from 20 (PRU-*WT*), 20 (PRU*ΔGRA15*) and 30 (PRU*ΔGRA15* + *GRA15*) cortical sections per condition from three independent experiments (PRU-*WT,* n=2; PRU*ΔGRA15,* n=2; PRU*ΔGRA15* + *GRA15* n=3 mice per condition).
E. Relative expression (qPCR) of *T. gondii* TgB1 gene (mean ± SEM) in brain tissue of mice inoculated with DC/*Tg* PRU-*WT versus* PRU*ΔGRA15.* Infected DCs ($2x10^4$DCs / $1x10^4$ cfu *Tg*) were inoculated in the ICA of mice in control (*Tg*), heparin or LPS-pre-treated conditions. Brains were extracted 7 dpi. Data from three independent experiments (n= 4 mice).
F. Experimental set up. Freshly egressed *T. gondii* (*Tg*) tachyzoites (PRU-GFP, $2x10^5$ cfu or RH-CPS-mCherry, $10x10^6$ cfu) or control medium were inoculated ip in mice. Brains were extracted 48 hpi and micro-vessels purified.
G. Relative mRNA expression (qPCR) of *Icam1* (ICAM-1), *Vcam1* (VCAM-1) and *Elam1* (e-selectin) in brain micro-vessels for the indicated conditions. Data are expressed as mean (± SEM) from three independent experiments (n= 3 mice).
Statistical analyses: (**C**) 2-tailed Unpaired student's t-test, (**D**) Kruskal-Wallis followed by Dunn's multiple comparison test. (**E**) One-way ANOVA followed by Bonferroni's multiple comparison test, numeric p values are indicated, ns: non-significant.
Source data are provided as a Source Data file.
