# Supplementary material for: ICAM-1/CD18-mediated sequestration of parasitized phagocytes in cortical capillaries promotes neuronal colonization by Toxoplasma gondii
Source: Nat Commun. 2025 Apr 14;16:3529. doi: 10.1038/s41467-025-58655-z (PMC11997185; doi:10.1038/s41467-025-58655-z)
Supplement: Supplementary file 2 — Description of Additional Supplementary Files [file 41467_2025_58655_MOESM2_ESM.pdf]

Description of Additional Supplementary Files

Movie S1. Sequestration of infected DC in the vascular branching point
Confocal micrograph shows the intravascular localization of *T. gondii* type I RH (GFP^+, green)-infected DCs (CMTMR, red) in relation to the vascular marker Evans blue (cyan). Infected CMTMR pre-labelled DCs ($20x10^6$ DCs / $10x10^6$ cfu *Tg*) were inoculated in the ICA and brains extracted at 16 hpi.

Movie S2. After egressing from the infected DC, tachyzoites transmigrate across the BBB
Confocal micrographs and corresponding 3D surface analysis show the localization of type I RH tachyzoites (GFP^+, green) and DCs remnant ("*ghost*", $CMTMR^+$, grey) in relation to the vascular marker Evans blue (red). Infected CMTMR pre-labelled DCs ($20x10^6$ DCs / $10x10^6$ cfu *Tg*) were inoculated in the ICA and brains extracted at 16 hpi.

Movie S3. *T. gondii* tachyzoites confined inside neuron after egressing from infected DC
Confocal micrographs and corresponding 3D surface analysis show the localization of type I RH tachyzoites (GFP^+, green) and neurons ($NeuN^+$, grey) in relation to the vascular marker Evans blue (red). Infected CMTMR pre-labelled DCs ($20x10^6$ DCs / $10x10^6$ cfu *Tg*) were inoculated in the ICA and brains extracted at 16 hpi.

Movie S4. *T. gondii* tachyzoite confined inside neuron after egressing from infected DC
Confocal micrographs and corresponding 3D surface analysis show the localization of GFP-expressing RH tachyzoites (GFP^+, green), DCs ($CMTMR^+$, blue) and neurons ($NeuN^+$, grey) in relation to the vascular marker Evans blue (red). Infected CMTMR pre-labelled DCs ($20x10^6$ DCs / $10x10^6$ cfu *Tg*) were inoculated in the ICA and brains extracted at 28 hpi.

Movie S5. *T. gondii* tachyzoite confined in the brain parenchyma
Confocal micrographs and corresponding 3D surfaces show the extravascular localization of type I RH wild type (GFP^+, green) tachyzoites in relation to the vascular marker Evans blue (red). $20x10^6$ cfu of freshly egressed tachyzoites were inoculated in the ICA and brains extracted 1 hpi.

Movie S6. Non-replicative *T. gondii* tachyzoite (CPS) confined in the brain parenchyma
Confocal micrographs and corresponding 3D surfaces show the extravascular localization of non-replicative type I RH-CPS ($mCherry^+$, red) tachyzoites in relation to the vascular marker Evans blue (grey). $20x10^6$ cfu of freshly egressed tachyzoites were inoculated via the ICA and brains were extracted 1 hpi.

Movie S7. *T. gondii*-infected DC confined in the brain parenchyma
Confocal micrograph shows the extravascular localization of *T. gondii* type II PRU (GFP^+, green)-infected DCs (CMTMR, red) in relation to the vascular marker Evans blue (cyan). $20x10^6$ DCs / $10x10^6$ cfu *Tg* were inoculated in the ICA of LPS pre-treated mice and brains extracted at 16 hpi.
