# Supplementary material for: ICAM-1/CD18-mediated sequestration of parasitized phagocytes in cortical capillaries promotes neuronal colonization by Toxoplasma gondii
Source: Nat Commun. 2025 Apr 14;16:3529. doi: 10.1038/s41467-025-58655-z (PMC11997185; doi:10.1038/s41467-025-58655-z)
Supplement: Supplementary file 10 — Reporting Summary [file 41467_2025_58655_MOESM10_ESM.pdf]

nature portfolio

Corresponding author(s): Antonio Barragan

Last updated by author(s): Mar 17, 2025

Reporting Summary

Nature Portfolio wishes to improve the reproducibility of the work that we publish. This form provides structure for consistency and transparency in reporting. For further information on Nature Portfolio policies, see our Editorial Policies and the Editorial Policy Checklist.

Statistics

For all statistical analyses, confirm that the following items are present in the figure legend, table legend, main text, or Methods section.

n/a	Confirmed	
☐	☒	The exact sample size (n) for each experimental group/condition, given as a discrete number and unit of measurement
☐	☒	A statement on whether measurements were taken from distinct samples or whether the same sample was measured repeatedly
☐	☒	The statistical test(s) used AND whether they are one- or two-sided<br>*Only common tests should be described solely by name; describe more complex techniques in the Methods section.*
☒	☐	A description of all covariates tested
☒	☐	A description of any assumptions or corrections, such as tests of normality and adjustment for multiple comparisons
☐	☒	A full description of the statistical parameters including central tendency (e.g. means) or other basic estimates (e.g. regression coefficient) AND variation (e.g. standard deviation) or associated estimates of uncertainty (e.g. confidence intervals)
☐	☒	For null hypothesis testing, the test statistic (e.g. F, t, r) with confidence intervals, effect sizes, degrees of freedom and P value noted<br>*Give P values as exact values whenever suitable.*
☒	☐	For Bayesian analysis, information on the choice of priors and Markov chain Monte Carlo settings
☒	☐	For hierarchical and complex designs, identification of the appropriate level for tests and full reporting of outcomes
☒	☐	Estimates of effect sizes (e.g. Cohen's d, Pearson's r), indicating how they were calculated

Our web collection on statistics for biologists contains articles on many of the points above.

Software and code

Policy information about availability of computer code

Data collection	ZEN blue 2.1 software. Leica LAS X Software. QuantStudio™ Design and Analysis Software. BD FACSDiva™ Software.
Data analysis	IMARIS v.10.1 software; Fiji/ImageJ software v.2.9.0/1.54f; FlowJo 10.10.0; GraphPad Prism v.10 software; QuantStudio™ Design and Analysis Software.

For manuscripts utilizing custom algorithms or software that are central to the research but not yet described in published literature, software must be made available to editors and reviewers. We strongly encourage code deposition in a community repository (e.g. GitHub). See the Nature Portfolio guidelines for submitting code & software for further information.

Data

Policy information about availability of data

All manuscripts must include a data availability statement. This statement should provide the following information, where applicable:

- Accession codes, unique identifiers, or web links for publicly available datasets
- A description of any restrictions on data availability
- For clinical datasets or third party data, please ensure that the statement adheres to our policy

Source data are provided in this paper. All data needed to support the conclusions are presented in the paper, the Supplementary Information and the Source Data file.

Research involving human participants, their data, or biological material

Policy information about studies with human participants or human data. See also policy information about sex, gender (identity/presentation), and sexual orientation and race, ethnicity and racism.

Reporting on sex and gender	N/A
Reporting on race, ethnicity, or other socially relevant groupings	N/A
Population characteristics	N/A
Recruitment	N/A
Ethics oversight	N/A

Note that full information on the approval of the study protocol must also be provided in the manuscript.

Field-specific reporting

Please select the one below that is the best fit for your research. If you are not sure, read the appropriate sections before making your selection.

☒ Life sciences ☐ Behavioural & social sciences ☐ Ecological, evolutionary & environmental sciences

For a reference copy of the document with all sections, see nature.com/documents/nr-reporting-summary-flat.pdf

Life sciences study design

All studies must disclose on these points even when the disclosure is negative.

Sample size	All experiments were performed with enough independent biological samples (a minimum of 3) to allow statistical analysis. For each experimental condition the sample size is described in the figure legend.
Data exclusions	No data exclusion.
Replication	All experiments were successfully replicated. For each experimental condition the number of biological replicates is indicated in the figure legend.
Randomization	For mouse experiments, sex and age-matched groups were used and mice were randomly attributed to the different conditions and, when possible, housed in the same animal cages during experiments.
Blinding	Experimental groups were blinded for mouse tissue analyses. Typically, one individual performed animal experimentation and 2-3 different individuals analyzed the experimental parameters in codified samples (1,2,3...). For in vitro cell culture assays (cell adhesion), blinding was not possible due to the need for labeling during culture required by Swedish BSL-2 organism regulations.

Reporting for specific materials, systems and methods

We require information from authors about some types of materials, experimental systems and methods used in many studies. Here, indicate whether each material, system or method listed is relevant to your study. If you are not sure if a list item applies to your research, read the appropriate section before selecting a response.

Materials & experimental systems

n/a	Involved in the study	
☐	☒	Antibodies
☐	☒	Eukaryotic cell lines
☒	☐	Palaeontology and archaeology
☐	☒	Animals and other organisms
☒	☐	Clinical data
☒	☐	Dual use research of concern
☒	☐	Plants

Methods

n/a	Involved in the study	
☒	☐	ChIP-seq
☐	☒	Flow cytometry
☒	☐	MRI-based neuroimaging

Antibodies

Antibodies used	Anti-mo CD54 (eBioscience, Cat# 14-0541-85, Clone YN1/1.7.4, Lot# 2089516), dose: 0,2 mg/kg. Anti-mo LFA-1 beta (eBioscience, Cat# 14-0181-85, Clone M18/2, Lot# 2577324), dose: 0,2 mg/kg. Isotype (eBioscience, Cat# 14-4321-85, Clone eBR2a, Lot# 2777082), dose: 0,2 mg/kg. Anti-mo CD45 Alexa fluor 647-conjugated antibody (R D Systems, Cat# FAB3507R, Clone 319211, Lot# 1726103), dose: 0,4 mg/Kg. PE-Cyanine7 anti-mo CD11c (eBioscience, Cat# 25-0114-82, Clone N418, Lot# 2142958), dilution: 1/50 (v/v). PE anti-mo CD18 (eBioscience, Cat# 101407, Clone M18/2. Lot# B313003), dilution: 1/50 (v/v). Super Bright 702 anti-mo MHCII I-A/I-E (eBioscience, Cat# 67-5321-82, Clone M5/114.15.2, Lot# 2020037), dilution: 1/50 (v/v). Anti-NeuN (Abcam, Cat# ab177487, Clone EPR12763, Lot# GR3275122-10), dilution: 1/250 (v/v). Anti-GPAF (Invitrogen, Cat# 13-0300, Clone 2.2B10, Lot# VH307339), dilution: 1/250 (v/v). Alexa Fluor 594-conjugated secondary antibodies (Invitrogen, Cat# A21442, Lot# 2906378; Molecular probes, Cat# A2147, Lot# 52521A), dilution: 1/1000 (v/v).
Validation	The references below provide additional information of the antibodies used in this study.<br><br>anti-NeuN<br>anti-GPAF<br>Anti-mo CD54<br>Olivera, G.C., Ross, E.C., Peuckert, C. & Barragan, A. Blood-brain barrier-restricted translocation of Toxoplasma gondii from cortical capillaries. Elife 10 (2021).<br><br>CD45-647<br>https://www.rndsystems.com/products/mouse-cd45-alexa-fluor-647-conjugated-antibody-319211_fab3507r#product-details<br><br>anti-mo LFA-1 beta<br>Ross, E.C., Hoeve, A.L.T., Saeij, J.P.J. & Barragan, A. Toxoplasma effector-induced ICAM-1 expression by infected dendritic cells potentiates transmigration across polarised endothelium. Front Immunol 13, 950914 (2022).<br><br>anti-mo CD11c<br>PE anti-mo CD18<br>anti-mo MHCII I-A/I-E<br>Ten Hoeve, A.L. et al. The Toxoplasma effector GRA28 promotes parasite dissemination by inducing dendritic cell-like migratory properties in infected macrophages. Cell host & microbe 30, 1570-1588 e1577 (2022).

Eukaryotic cell lines

Policy information about cell lines and Sex and Gender in Research

Cell line source(s)	Murine brain endothelial cell line bEnd.3 (ATCC, CRL-2299, unknown sex); Human foreskin fibroblast line HFF (ATCC, CRL-2088, sex male).<br>Toxoplasma gondii cell lines: RH-GFP, RHΔTgWIP-GFP, RH CPS-mCherry, ME49-RFP, PRU-GFP, PRUΔTgGRA15-GFP, PRUΔTgGRA15+GRA15-GFP and CTG (ATCC, 50842).
Authentication	bEnd.3, HFFs and T. gondii CTG cell lines were purchased from ATCC. No authentication procedure was performed.<br><br>Toxoplasma gondii cell lines: RH-GFP, RHΔTgWIP-GFP, RH CPS-mCherry, ME49-RFP, PRU-GFP, PRUΔTgGRA15-GFP, PRUΔTgGRA15+GRA15-GFP were not authenticated.<br><br>RH-GFP, RHΔTgWIP-GFP, PRU-GFP, PRUΔTgGRA15-GFP, PRUΔTgGRA15+GRA15-GFP and ME49-RFP cells lines were provided by Prof. Jeroen P J Saeij, UC Davis.<br>-Sangare, L.O. et al. In Vivo CRISPR Screen Identifies TgWIP as a Toxoplasma Modulator of Dendritic Cell Migration. Cell Host Microbe 26, 478-492 e478 (2019).<br>-Wang, Y. et al. Three Toxoplasma gondii Dense Granule Proteins Are Required for Induction of Lewis Rat Macrophage Pyroptosis. mBio 10 (2019).<br>-Rosowski, E.E. et al. Strain-specific activation of the NF-kappaB pathway by GRA15, a novel Toxoplasma gondii dense granule protein. J Exp Med 208, 195-212 (2011).<br>- Rosowski, E.E., D. Lu, L. Julien, L. Rodda, R.A. Gaiser, K.D. Jensen, and J.P. Saeij. 2011. Strain-specific activation of the NF-kappaB pathway by GRA15, a novel Toxoplasma gondii dense granule protein. The Journal of experimental medicine 208:195-212<br><br>RH CPS-mCherry parasites were provided by Prof. David Bzik and Prof Chris Hunter, U Pennsylvania.<br>Fox, B.A. & Bzik, D.J. De novo pyrimidine biosynthesis is required for virulence of Toxoplasma gondii. Nature 415, 926-929 (2002).
Mycoplasma contamination	All cell lines used in this study were routinely tested for mycoplasmas by qPCR every 4 months. All cell line were negative for mycoplasma.
Commonly misidentified lines<br>(See ICLAC register)	No commonly misidentified lines were used in this study.

Animals and other research organisms

Policy information about studies involving animals; ARRIVE guidelines recommended for reporting animal research, and Sex and Gender in Research

Laboratory animals	All experiments were performed using male and female C57BL/6NCrl mice (strain code 027, Charles River), aged 4 to 10 weeks, housed in a ventilated facility, provided with unrestricted access to tap water and food, and kept under a 12-hour light/dark cycle at a temperature of 20–22 °C.
Wild animals	No wild animals were used in this study.
Reporting on sex	Both female and male animals were used in this study.
Field-collected samples	No field-collected samples were used in this study
Ethics oversight	The Regional Animal Research Ethical Board, Stockholm, Sweden, approved experimental procedures in mice and protocols involving extraction of cells from mice (permit number 16403-2022), following proceedings described in EU legislation (Council Directive 2010/63/EU). All methods were carried out in accordance with relevant guidelines and regulations. All methods are reported in accordance with ARRIVE (Animal Research: Reporting of In Vivo Experiments) guidelines (https://arriveguidelines.org).

Note that full information on the approval of the study protocol must also be provided in the manuscript.

Plants

Seed stocks	N/A
Novel plant genotypes	N/A
Authentication	N/A

Flow Cytometry

Plots

Confirm that:

☒ The axis labels state the marker and fluorochrome used (e.g. CD4-FITC).

☒ The axis scales are clearly visible. Include numbers along axes only for bottom left plot of group (a 'group' is an analysis of identical markers).

☒ All plots are contour plots with outliers or pseudocolor plots.

☒ A numerical value for number of cells or percentage (with statistics) is provided.

Methodology

Sample preparation	For blood samples.<br>Blood was collected by cardiac puncture into heparinized tubes and peripheral blood mononuclear cells (PBMCs) were purified with LymphoprepTM (STEMCELL Technologies, Cat# 07801). Cells were fixed in 4% PFA (Histolab, Cat# 02176), resuspended in FACS buffer.<br><br>For infected dendritic cells.<br>DCs were challenged with freshly egressed tachyzoites for 5 h, washed in PBS and stained in FACS buffer (0,5% BSA, 2mM EDTA in PBS). Challenged DCs were stained with PE-Cyanine7 anti-mo CD11c PE anti-mo CD18 and Super Bright 702 anti-mo MHCII I-A/I-E antibodies and immediately analyzed.
Instrument	LSR Fortessa
Software	BD FACSDiva™ Software. FlowJo 10.10.0
Cell population abundance	Abundant.<br><br>Purity from blood samples (Fig. 1 and Fig. S1) was determined by detection of CD45 positive cells.

Purity of the population of interest in Fig. S4 was determined by the expression level of CD11c and MHCII.

Gating strategy

Gating based for FSC-A/SSC-A, singlets and CD45 expression is exemplified in Fig. S1A. Gating for CD11c and MHCII expression has been published in Ross et al., Cell Mol Life Sci, 2021 (Fig. S2).

☒ Tick this box to confirm that a figure exemplifying the gating strategy is provided in the Supplementary Information.
